# New Drugs and Preparations

**Published:** 1894-12-29

**Authors:** 


### New Drugs and Preparations.

[We shall be glad to receive, at our Office, 428, Strand, London, W.C., from the manufacturers, specimens of all new preparations and drugs which may be brought out from time to time.]

### POISONING BY ACONITINE.

A druggist swallowed by mistake a pill containing a fraction over one-fifth grain of crystalline aconitine. Almost immediately after an emetic was taken and the stomach washed out. The first symptom noted was a sensation of burning in the mouth, and the same in the face and extremities. Forty minutes after the taking of the poison the face was pale, the voice was almost lost, there was a feeling of impending death, the pulse was very small, rapid, and difficult to count, and the respiration was feeble and irregular. Coldness of the extremities, clammy perspiration, collapse, and loss of consciousness supervened. At this time 15 grains of caffeine and 45 minims of ether were injected subcutaneously, and hot-water bottles and rubbing freely used to the surface of the body. The pulse was now between 40 and 50 per minute, and iced champagne was freely administered. Two hours later the collapse was more marked, the pulse was 30 per minute and scarcely perceptible, the respiration almost gone, the pupils widely dilated, and there was frequent vomiting and muscular tremors. Fifteen grains caffeine and half a drachm of sulphuric ether were again given subcutaneously, with improvement in the symptoms, and seven-grain doses of caffeine were several times administered at intervals. After nine hours the severe symptoms gradually disappeared, but for several days there was great prostration, and at the end of a week the patient had not entirely recovered. The valuable result obtained by the use of large doses of caffeine is of interest.—(*Therap. Gazette*, February, 1894.)

### LARGE DOSES OF MORPHINE.

In the *Lancet* of March 17th, 1894, a case is recorded in which a servant girl, aged nineteen, unaccustomed to opium in any form, administered to herself hypodermically about 12 grains of acetate of morphine. In two or three minutes afterwards she was narcotised, and the symptoms were very pronounced, there being twice complete stoppage of the respiration, total absence of the conjunctival reflex for some hours, and pin-point pupil. She was treated with atropine. galvanism, artificial respiration, coffee, &c., and at the end of twelve hours the symptoms had become less urgent, but even after thirty-six hours, unless treatment was kept up, the respiration threatened to stop and cyanosis rapidly developed.

In the *Medical Record* of September 15th, 1894, Dr. Groom reports a case of a woman, a habitual morphine consumer, to whom he administered 15 grains of morphine hypodermically at one dose for the relief of pain and vomiting. The woman herself stated that she was accustomed to take about 30 grains daily by the mouth in divided doses.

